# Dynamics of the Equine Placental DNA Methylome and Transcriptome from Mid- to Late Gestation

**DOI:** 10.3390/ijms24087084

**Published:** 2023-04-11

**Authors:** Daniela Orellana-Guerrero, José M. Uribe-Salazar, Hossam El-Sheikh Ali, Kirsten E. Scoggin, Barry Ball, Peter Daels, Carrie J. Finno, Pouya Dini

**Affiliations:** 1 Department of Population Health and Reproduction, School of Veterinary Medicine, University of California, Davis, CA 95616, USA; 2 Genome Center, University of California, Davis, CA 95616, USA; 3 Gluck Equine Research Center, Department of Veterinary Science, University of Kentucky, Lexington, KY 40546, USA; 4 College of Veterinary Medicine, Mansoura University, Mansoura 35516, Egypt; 5 Faculty of Veterinary Medicine, Ghent University, 9820 Merelbeke, Belgium

**Keywords:** chorioallantois, methylation, reduced representation bisulfate sequencing, differentially methylated regions, horse, placental methylome

## Abstract

The placenta is a temporary organ that is essential for the survival of the fetus, with a lifelong effect on the health of both the offspring and the dam. The functions of the placenta are controlled by its dynamic gene expression during gestation. In this study, we aimed to investigate the equine placental DNA methylome as one of the fundamental mechanisms that controls the gene expression dynamic. Chorioallantois samples from four (4M), six (6M), and ten (10M) months of gestation were used to map the methylation pattern of the placenta. Globally, methylation levels increased toward the end of gestation. We identified 921 differentially methylated regions (DMRs) between 4M and 6M, 1225 DMRs between 4M and 10M, and 1026 DMRs between 6M and 10M. A total of 817 genes carried DMRs comparing 4M and 6M, 978 comparing 4M and 10M, and 804 comparing 6M and 10M. We compared the transcriptomes between the samples and found 1381 differentially expressed genes (DEGs) when comparing 4M and 6M, 1428 DEGs between 4M and 10M, and 741 DEGs between 6M and 10M. Finally, we overlapped the DEGs and genes carrying DMRs (DMRs-DEGs). Genes exhibiting (a) higher expression, low methylation and (b) low expression, high methylation at different time points were identified. The majority of these DMRs-DEGs were located in introns (48.4%), promoters (25.8%), and exons (17.7%) and were involved in changes in the extracellular matrix; regulation of epithelial cell migration; vascularization; and regulation of minerals, glucose, and metabolites, among other factors. Overall, this is the first report highlighting the dynamics in the equine placenta methylome during normal pregnancy. The findings presented serve as a foundation for future studies on the impact of abnormal methylation on the outcomes of equine pregnancies.

## 1. Introduction

The placenta is the feto–maternal interface that plays a central role in the health of both the fetus and the mother [1,2,3]. This temporary organ is not only essential for nutrients, gas, and waste exchange between fetal and maternal circulation, but it also produces several hormones and growth factors, protects the fetus from the maternal immune system, and regulates the intrauterine environment [1,2,4,5,6]. The placentae of all eutherian mammals provide common structural and functional features; however, there are striking differences between species in the gross and microscopic structure of the placenta, including the number of tissue layers between the maternal and fetal vascular systems and the degree of interconnections between the fetal and maternal components of the placenta [7,8].

Horses (*Equus caballus*) have chorioallantoic placentae, in which the chorionic surface forms microvilli that are juxtaposed to the endometrial epithelium with minimal extension into the uterine mucosa [7,8,9,10]. Therefore, the fetal placenta (chorioallantoic membrane (CA)) can be separated from the endometrium with negligible cellular mixture from maternal components [9,10,11,12,13]. This feature of the equine placenta makes it an ideal model for evaluating the fetal part of the placenta without maternal cell residue, in contrast to the human and murine placentae, where, due to the type of placentation, separation of the fetal and maternal compartments represents a technical challenge [14]. Additionally, the equine placenta plays an important role in both gonadotrophin (equine chorionic gonadotrophin (eCG)) production at the beginning of gestation [15,16] and steroid production from ~80 days to term [10,17,18]. These unique features of the equine placenta are controlled by dynamic gene expression and genetic regulatory mechanisms throughout the gestational process [12,19].

Within the last few years, several studies have demonstrated the dynamics of gene expression in the equine placenta [11,12,19,20]. Multiple transcripts associated with endocrine and immune function, angiogenesis, iron-binding proteins, extracellular matrix proteins, transport proteins, and antioxidants have been found to be differentially expressed over the course of gestation [12,19,21,22]. The parental bias in the expression of these genes has also been investigated, demonstrating that paternally expressed genes are mainly involved in metabolic and biosynthesis processes of proteins, macromolecules, and organic compounds, while maternally expressed genes are involved in the positive regulation of cell death and apoptotic processes [9]. However, the underlying mechanisms that control the gene expression in the equine placenta have not been investigated.

In mammals, transcriptional regulation involves multiple epigenetic mechanisms, which are constant heritable chemical and conformational modifications of DNA or its associated histone proteins that do not affect the nucleotide sequence per se [6,23,24,25]. One widely studied epigenetic modification is DNA methylation, which consists of an addition of a methyl group (-CH3) to the cytosine bases of DNA, which are then transformed to 5-methylcytosine by DNA methyltransferase enzymes (DNMTs) [3,4,6,23,24,25,26,27,28]. DNA methylation has been identified as being required for several biological processes, such as embryonic development, X-chromosome inactivation, cell differentiation, and genomic imprinting [27,29]. This epigenetic mark is widespread in the DNA and remains constant from generation to generation, particularly in the case of CpG islands that consist of ~1 kilobase (kb) of high cytosine-guanine (C-G) content. These CpG islands tend to be unmethylated when close to a gene promoter and can repress transcription directly by blocking the binding of transcription factors when methylated or indirectly by using CpG binding protein that has a suppressing effect on chromatin remodeling [4,6,23,24,25,26,27,28]. CpG islands are commonly found close to transcription start sites, showing that these might be a detection pattern for the gene expression [27,29,30]. Overall, it has been suggested that DNA methylation represses gene expression, perhaps by blocking the promoters at which activating transcription factors should bind [4,5,29,31]. Nevertheless, the exact impact of this epigenetic regulation on the gene expression patterns across different mammalian tissues is not completely understood.

It has been reported that the human placenta has a distinctive methylome that is characterized by high variability in DNA methylation compared to other tissues and by being globally hypomethylated [4,6,25,26,28,31,32,33]. Moreover, it has been elucidated that alterations in the DNA methylation pattern are associated with abnormal gene expression and many pathologies in utero and in extrauterine life [24,27,31,33,34]. Dysregulation of the placental DNA methylation patterns in human and mouse pregnancies can have deleterious consequences which can affect the normal placental development (i.e., placental insufficiency and intrauterine growth restriction (IUGR)) [6,9,23,24]. In addition, the placental methylome links environmental factors and placental pathologies, affecting fetal growth and adult life [4]. Several pathological conditions can affect pregnancy outcomes in mares, with a majority of these conditions manifesting in mid–late-term gestation. Therefore, detailing the equine placenta methylome during these stages of gestation is vital for a proper understanding of the dynamic processes of gene regulation throughout gestation and how these changes can affect the outcome of the pregnancy.

We hypothesized that the equine placenta—the horse being a species with minimal maternal residue in the fetal placenta—has a dynamic and distinct methylome pattern throughout gestation, regulating the gene expression and functioning of this transient organ. Therefore, the aims of our study were to identify the dynamics of the equine placenta methylome from mid- to late gestation and to evaluate the effect of the placental methylome on placental gene expression. Our ultimate goal was to map the landscape of the placental DNA methylome during normal gestation to understand the physiological mechanisms of placental gene expression without biases related to maternal cellular contamination. This information will further build a foundation for future studies investigating the effects of altered placental DNA methylation during pregnancy pathologies.

## 2. Results

### 2.1. Methylation Patterns in the Equine Placenta

**Figure 1:**
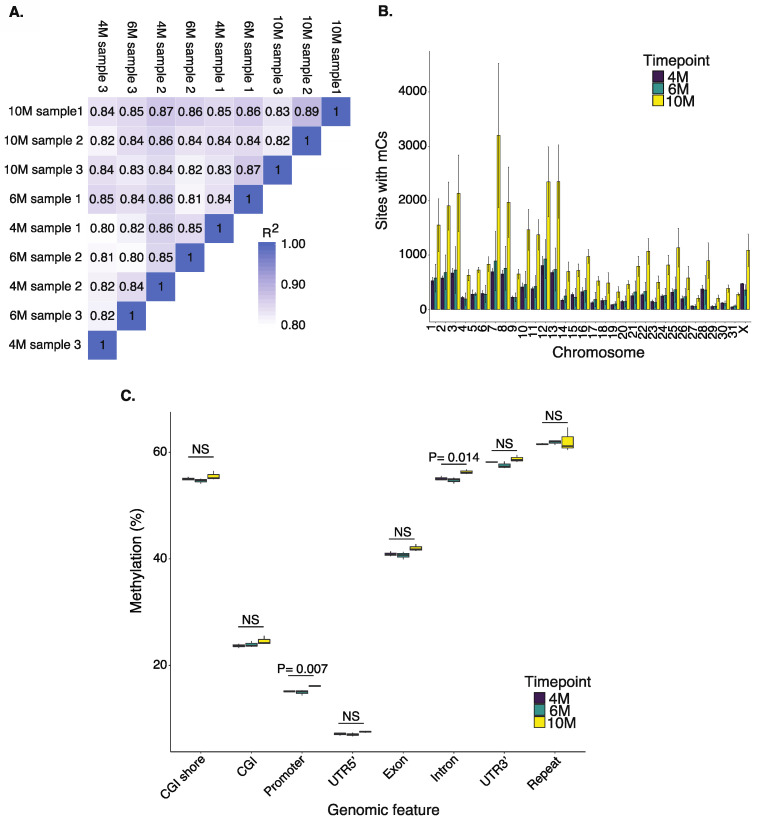
Descriptive representation of mCs in the equine placenta at 4M, 6M, and 10M in the CG context. (**A**) Correlation of the detected mCs among the samples. (**B**) Distribution of mCs shows a similar pattern across chromosomes. (**C**) Description of the methylation levels in the genomic functional regions. All true methylated cytosine sites were annotated to the reference equine transcriptome. NS: no significant difference (*p* > 0.05).

Equine chorioallantois (CA) samples were collected at four (4M), six (6M), and ten months (10M) of gestation (three samples at each time point), and DNA was extracted from the samples, followed by reduced representation bisulfite sequencing (RRBS; Material and Methods Section). On average (±SD), 42.3 ± 9.3 million reads were generated per sample with >93% with quality > Q30 (93.51% for 4M, 93.49% for 6M, and 93.89% for 10M) and >99.41% bisulfite conversion rate (Supplementary Table S1). On average, 52 ± 5% of reads were uniquely mapped to the reference genome, and all time points exhibited a similar number of methylated cytosines (mCs) (Supplementary Table S1). The detected mCs were predominantly observed in a CG context, encompassing >99% of the total mCs at 4M, >98% at 6M, and >97% at 10M (Supplementary Table S1). All samples exhibited high correlations in the total number of mCs identified in the CG context (average Pearson r > 0.80 and *p* < 0.01; Figure 1A). The information related to the non-CG context is available in Supplementary Figure S1A,B.

The distribution of mCs at all time points revealed a similar pattern across chromosomes (Figure 1B). The distribution of mCs at 4M was weakly correlated with chromosome sizes (*p* = 0.033, R^2^ = 0.11), where larger chromosomes housed more mCs. At 6M and 10M, the mC distributions did not exhibit associations with chromosome size (6M: *p* = 0.055, R^2^ = 0.09; 10M: *p* = 0.070, R^2^ = 0.08).

### 2.2. Dynamics of DNA Methylation during Gestation

To identify the methylation levels in functional genomic regions, all true methylated cytosine sites (according to the binomial tests; see Section 4) were annotated to the reference transcriptome. Among the eight evaluated types of genomic features (CpG islands (CGI), CGI shores, promoters, UTRs at 3′ and 5′, exons, introns, and repeats), the lowest methylation percentages were found in UTRs at the 5′ end, followed by promoters and CGIs for mCs in the CG context (*p* < 0.001; Figure 1C). After detailing the methylation percentages of the different genomic features between developmental time points (4M, 6M, and 10M), it was identified that methylation in promoters (*p* = 0.007) and introns (*p* = 0.014) was higher at 10M than at the earlier time points (Figure 1C, Supplementary Table S2).

**Figure 2:**
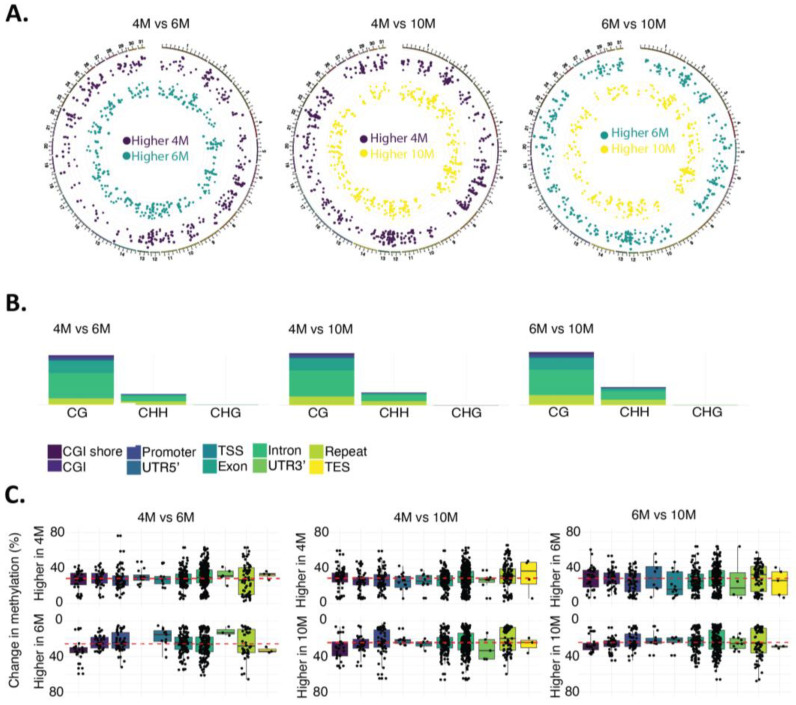
Differentially methylated regions (DMRs) across gestational times. (**A**) DMR distribution across chromosomes. (**B**) Distribution of DMRs according to the contexts and genomic features. The majority of DMRs were identified in the CG context and landed in introns (~40%) and exons (~20%), followed by repeats (~11%) and promoters (~10%). (**C**) Methylation changes in DMRs across the time points and among the genomic features. An ~30% change (median) in methylation percentages on the DMRs was noted across all genomic features, represented by the pink dashed lines in the figure.

Next, we compared the methylation levels across the developmental time points to identify differentially methylated regions (DMRs) using a Bayesian hierarchical model and Wald tests with dispersion shrinkage (DSS). A total of 921 DMRs were identified between 4M and 6M, 1225 DMRs between 4M and 10M, and 1026 DMRs between 6M and 10M samples. The length of the DMRs remained constant among samples, with ~97% of the DMRs having a length ≤ 200 bp (median length: 107 bp) (Supplementary Figure S2). On average, 23.1 mCs resided within each DMR, ranging from 4 to 1115 sites. DMRs were identified in all chromosomes between all compared time points, with no difference in the density of DMRs across chromosomes (Figure 2A).

The number of DMRs localized in annotated genes was 817 comparing 4M and 6M (454 DMRs with higher methylation at 4M and 363 DMRs with higher methylation at 6M), 978 comparing 4M and 10M (553 DMRs with higher methylation at 4M and 425 DMRs with higher methylation at 10M), and 804 comparing 6M and 10M (430 DMRs with higher methylation at 6M and 374 DMRs with higher methylation at 10M; Supplementary Table S3). As expected, the majority of DMRs were identified in the CG context (4M vs. 6M: 83.5%, 4M vs. 10M: 81.6%, 6M vs. 10M: 76.5%; Figure 2B). The majority of DMRs intersecting gene features landed in introns (~40%) and exons (~20%), followed by repeats (~11%) and promoters (~10%) (Figure 2B, Supplementary Table S3). Next, we evaluated the percentage differences in methylation from the identified DMRs between time points for the different genomic features. Overall, we observed an ~30% change in methylation percentages in the DMRs across all genomic features (Figure 2C). This ~30% methylation difference in the DMRs identified remained constant in all comparisons (4M vs. 6M, 6M vs. 10M, 4M vs. 10M; Figure 2C).

**Figure 3:**
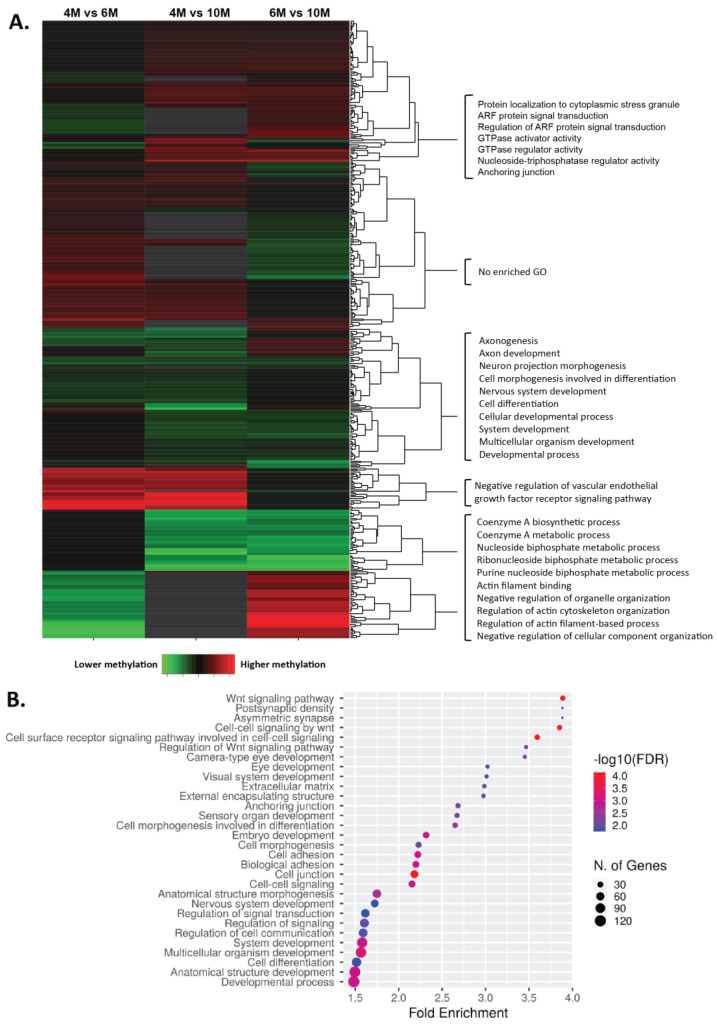
Differentially methylated genes and their gene ontologies among different gestational ages. (**A**) A total of 452 genes were differentially methylated in at least two of the comparisons (4M vs. 6M, 6M vs. 10M, 4M vs. 10M). (**B**) The GO analysis for these groups of genes revealed their enrichment in Wnt signaling, the developmental process, cell communication, and the extracellular matrix.

Of the 817 genes harboring DMRs between 4M and 6M, 205 have several DMRs in different regions of them, summing a total of 612 unique genes with DMRs (333 genes with higher methylation levels at 4M and 279 genes with higher methylation levels at 6M). Consecutively, 739 unique genes were identified between 4M and 10M (425 genes had higher methylation levels at 4M and 314 genes had higher methylation levels at 10M), and a total of 621 unique genes between 6M and 10M (335 genes had higher methylation levels at 6M and 286 genes had higher methylation levels at 10M). Using these gene lists, we performed gene ontology (GO) analyses to reveal biological processes or molecular functions enriched in genes with DMRs between placental developmental time points (Supplementary Figure S3). Comparing 4M to 6M, the enriched GO terms were involved in several developmental processes, including vascular endothelial growth factor receptor signaling, extracellular matrix organization (higher methylation at 4M), cell differentiation, and biological adhesion (higher methylation at 6M). When comparing 4M and 10M, the enriched GO terms were involved in the regulation of cell communication (higher at 4M) and cell adhesion (higher at 10M), among others. Lastly, genes with DMRs between 6M and 10M were enriched in Wnt signaling (higher methylation at 6M) and chemotaxis (higher at 10M), among others. A total of 19 GO terms were identified to be common within the comparisons, including cell differentiation, cell signaling, developmental process, extracellular matrix organization, GTPase activator activity, and regulation of cell communication. Furthermore, a total of 452 genes exhibited DMRs in at least two of the comparisons (4M vs. 6M, 6M vs. 10M, 4M vs. 10M; Figure 3A). The GO analysis for these groups of genes revealed their enrichment in Wnt signaling, the developmental process, cell communication, and the extracellular matrix (Figure 3B).

### 2.3. Impact of Placental DNA Methylation on Gene Expression

**Figure 4:**
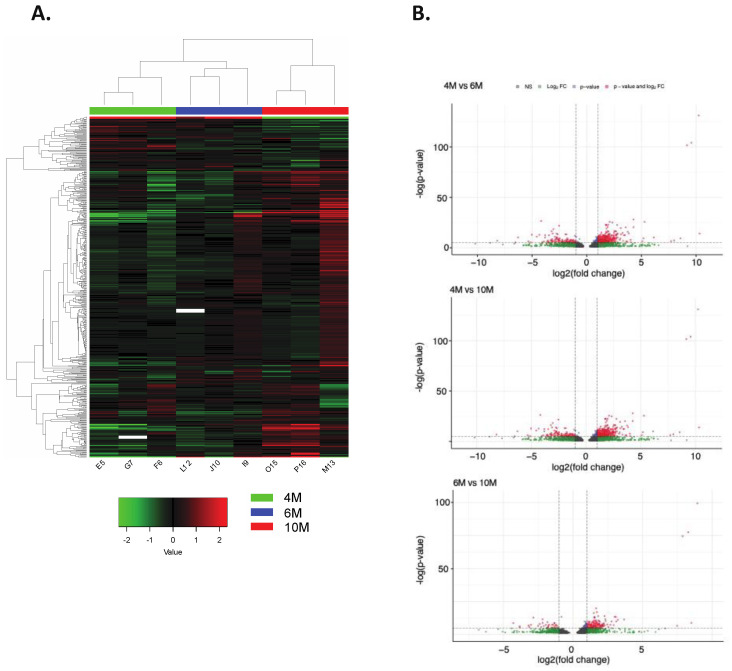
Differentially expressed genes among different gestational time points. (**A**) Heatmap of gene expression in chorioallantois from 4M, 6M, and 10M of gestation. (**B**) Differentially expressed genes were demonstrated using volcano plots.

**Figure 5:**
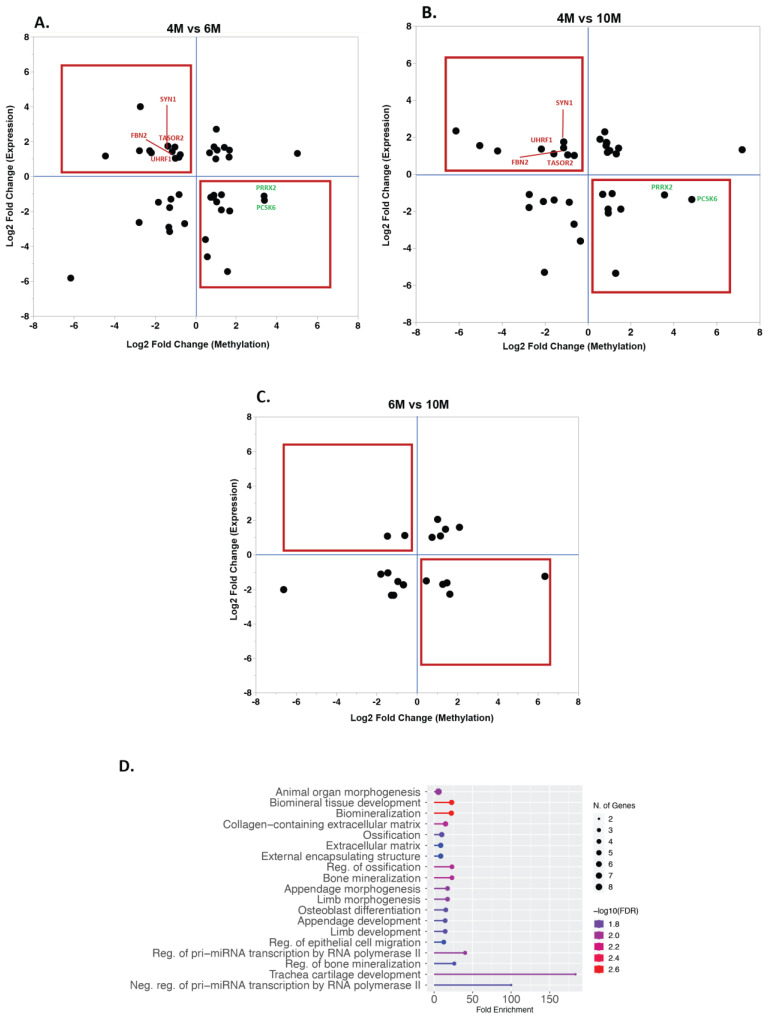
Association between gene expression and methylation. Based on the assumption that methylation influences gene expression, we identified the genes with reduced methylation that had an increased expression and genes with increased methylation that had a decreased expression ((**A**): 4M vs. 6M, (**B**): 4M vs. 6M, and (**C**): 6M vs. 10M; red squares indicate the genes which followed the expected patterns). (**D**) Gene ontology analysis of the 38 genes displaying accordance between methylation of DMRs and gene expression.

To assess the effect of methylation on gene expression, we evaluated previously generated RNA-sequencing data from the same samples. Reads were downloaded (GSE108279), mapped, and quantified to obtain differentially expressed genes (DEGs) (see Material and Methods). A total of 1381 DEGs (Bonferroni-adjusted *p*-value < 0.05 and log2(fold change) >1 or <−1) were found when comparing 4M and 6M samples (871 genes with higher expression at 4M; 510 genes had higher expression at 6M), 1428 DEGs between 4M and 10M samples (899 with higher expression at 4M; 529 with higher expression at 10M), and 741 DEGs between 6M and 10M samples (455 with higher expression at 6M; 286 with higher expression at 10M; Figure 4 and Supplementary Table S4). Next, we identified the overlap between the list of DEGs and genes carrying DMRs (Table 1), and, based on the assumption that methylation can have an effect on gene expression [25], we tested whether genes with reduced methylation had an increased expression and genes with increased methylation had a decreased expression (Figure 5A–C). We found 11 genes (*ANKRD44, ATXN1, BMPR1A, FBN2, LAMC3, MCC, PLIN1*, *SETBP1, SYN1, TASOR2*, and *UHRF1*) which had a higher expression at 4M than at 6M while having a lower methylation level at 4M. Moreover, we identified 10 genes (*ADAM33*, *GPR146, HSF4, IRX3, LMOD1, OBSCN, PCSK6, PRRX2, PTPRR,* and *SOX9*) with a lower expression and higher methylation level at 4M in comparison to 6M. Comparing 4M and 10M samples revealed eight genes (*FBN2*, *ILDR2, MALT1*, *PPARA*, *SYN1*, *TASOR2*, *UHRF1*, and *ZDBF2*) with a higher expression and lower methylation level at 4M than at 10M. A total of seven genes (*CPT1A*, *DHRS3*, LGSN, *PCSK6*, *PRRX2, RAMP1,* and *RNF17*) showed a lower expression and a higher methylation level at 4M than at 10M. Moreover, between 6M and 10M samples, one gene (*PTPRB*) had a higher expression and lower methylation level at 6M than at 10M, while five genes (*DES, RSPO2, SMOC2, SLC15A1*, and *TTC22*) had a lower expression and higher methylation at 6M than at 10M. Interestingly, *UHRF1, TASOR2, FBN2*, and *SYN1* showed high expression and low methylation at the earliest time point (4M) when compared to any of the other time points (6M or 10M), and *PCSK6* and *PRRX2* showed a lower expression and higher methylation at 4M than at 6M and 10M.

We further analyzed the genomic locations of DMRs for the genes for which there was an accordance between methylation status and expression level (n = 63, located on 39 unique genes; Figure 5). The majority of these DMRs were located in introns (48.4%), followed by promoters (25.8%) and exons (17.7%). Additionally, we combined all 38 genes displaying accordance between methylation of DMRs and gene expression data and found several GO terms involved in the collagen-containing extracellular matrix, extracellular matrix, regulation of epithelial cell migration, and regulation of pri-miRNA transcription, among others (Figure 5D).

## 3. Discussion

The goal of the current study was to profile the equine placental methylome during mid- to late gestation. The CA samples from four, six, and ten months of gestational age were analyzed to obtain a comprehensive methylation profile of the equine placenta. With a similar mapping rate to previous reports for RRBS of placentae (52.53% in this study vs. ~59–82% in the human placenta [35,36]), we evaluated the amount of mCs in different methylation contexts (CG, CHH, and CHG). Similar to previous reports, mCs were predominantly identified in the CG context [26,37,38]. The changes in the methylation patterns in the placenta are believed to be associated with placental function and might serve as a link between environmental factors and placental pathologies affecting fetal growth and well-being. In this study, we found a similar overall methylation percentage in the CA collected at four months and six months of gestation, with an increase in the methylation level at ten months of gestation in promoter and intron regions. These changes could be attributed to the maturity of the placenta as it reaches its final size and to villi density by mid-gestation (4–6 months of gestation) in mares [20], followed by preparation for parturition toward the end of the last trimester [12].

To further investigate the possible role of methylation dynamics in the equine placenta, we annotated the methylated sites and investigated the genes harboring differential methylation at each time point. We found that genes exhibiting significant methylation changes between gestational stages were enriched in several pathways, including vascular formation, extracellular matrix organization, cell adhesion, cell migration, and cell signaling, among others, playing indispensable roles in placental development and function [39]. Wnt signaling was one of the pathways that consistently showed enrichment in genes carrying DMRs in all comparisons between the studied stages of gestation, which is known to be essential in tissue homeostasis and cell proliferation, survival, and differentiation [40]. In humans, comparisons between placentae at different gestational stages showed a dynamic expression of the Wnt-β-catenin pathway [5,41]. Changes in the expression of Wnt signaling genes in human placentae have been linked to decreases in β-catenin in the later gestational stages, which could contribute to reduced placental invasiveness [41,42]. Moreover, studies in mouse placentae using single-cell RNA-seq have shown that Wnt plays a critical role in the establishment of cell heterogeneity in mid-gestation [43]. Additional reports in mice have evidenced the impact of that alteration in Wnt signaling genes in placental development, where deletions of *Wnt2* resulted in damaged vasculogenesis and *Wnt7b* knockout mice showed defective chorioallantoic fusion [44,45]. The identified dynamics of Wnt signaling in our equine placentae are concordant with previous reports that highlighted Wnt as a critical pathway in placental and embryonic development. Future studies are warranted to disentangle its role and implications in equine placental development, functions, and pathological conditions.

We found ~10% of the DMRs landed in promoters, highlighting their potential regulatory effect in equine placental gene expression dynamics. It is important to note that annotations in the equine genome are limited, and efforts are ongoing to improve these [46]. Existing reports have evidenced the impact of DNA methylation at promoters on gene regulation during placentation [47,48]. A significant portion of the DMRs intersecting genes were found in the intron regions (~40%), which is concordant with studies on human placentae [49]. Recent studies have shown that introns can carry regulatory sequences that impact gene expression [50,51,52]. Therefore, the DMRs located in genes are present in potential regulatory regions, emphasizing the importance of understanding their role in gene expression. Among the genes that showed accordance between their methylation status and expression pattern, 48.4% had their DMRs in intronic regions (Figure 5). Additionally, our results indicate that there is a trend of methylation increasing toward the end of gestation in promoters and introns, potentially due to changes in the regulatory networks during different stages of placental development.

Coupling our methylation data with the gene expression data from equine placentae allowed us to further investigate the possible role of methylation in equine placental development. The majority of DEGs carried their respective DMRs in introns (53.4%) and exons (24.1%) (Table 1). Therefore, we classified the genes that we found to be differentially expressed as decreased methylation/increased gene expression and increased methylation/decreased gene expression. Notably, there are more potential interactions between methylation and gene expression that could exist (e.g., gene body methylation increasing gene expression [Table 1]) that can be further studied and were not considered in this initial analysis. Since understanding these dynamics in detail was of paramount importance to us as we aim to develop a gene expression baseline throughout pregnancy to use as a reference for high-risk pregnancies or disease, we detailed the genes found in these different groups across developmental time points. One gene that evidenced increased expression and low methylation at 4M compared to the other stages (6M and 10M) was Synapsin I (*SYN1)*, a gene found to be related to mediating cell-to-cell fusion to form the syncytiotrophoblasts of placentae [53], which showed altered expression and methylation associated with fetal growth impairment [53]. In mice, the absence of expression of the *Syn1* orthologue gene leads to the death of pups in utero due to the inability of the syncytial layer to be established [53]. Likewise, in humans [54,55,56,57,58], it has been proposed that *SYN1* is important for placental development since its altered methylation and expression are related to fetal growth abnormalities. Makaroun and Himes [53] reported lower placental methylation in the regulatory regions of the *SYN1* gene along with an increase in its expression when comparing human fetuses presenting fetal growth restriction and small-for-gestational-age patients with a control group [53]. Evaluating the expression pattern of this gene in the equine abnormal placenta could potentially enable the identification of underlying causes of some of these conditions, such as growth-retarded foals or hydrops cases, which eventually could lead to the discovery of a biomarker for these placental pathologies.

The Fibrillin 2 *(FBN2)* gene presented the same methylation pattern (increased expression/low methylation at 4M vs. later stages). This gene encodes for an asprosin-like peptide hormone and has been found to be highly expressed in the human placenta, being called “placensin” [59]. In contrast, this gene is expressed in low amounts in murine placentae [59]. This peptide hormone is secreted by trophoblasts and seems to be involved in the formation of syncytiotrophoblasts and to lead to placental invasiveness [59]. Likewise, it could potentially stimulate cAMP release, glucose regulation, and gluconeogenesis, making this gene and its hormone vital to maintaining metabolic functions during human pregnancy [59]. This hormone seems to have gluconeogenic functions and could have an endocrine link to circulatory glucose increased production during human pregnancy [59]. During equine gestation, there is a profound change in the maternal circulatory glucose level to meet the high level of glucose demand in the foal [60]. This alteration in the glucose level was suggested to be associated with peripheral insulin resistance [61]. However, the effect of *FBN2* gene on the regulation of gluconeogenesis has not been studied and requires further investigation.

Protein Tyrosine Phosphatase 4A3 (*PTP4A3*) also displayed increased expression and low methylation at 4M. The family of protein tyrosine phosphatases (PTPs) has been associated with angiogenesis through signaling in vascular cells or by direct dephosphorylation of the Vascular Endothelial Growth Factor Receptors-2 (*VEGFR-2*s) [62]. *PTP4A3* expression has been associated with angiogenic processes in tumors, embryonic blood vessel expression, and expression in endothelial cells [62,63,64,65]. This gene has been reported to be involved in tumor angiogenesis, and its high expression was observed in mouse tumor endothelia, indicating its possible relation to the pathological angiogenesis necessary for tumor development and metastasis [65,66,67]. Cells lacking the gene were less invasive and migratory and deficient in gap closure [65]. Likewise, the presence of the *PTP43A* protein has been encountered only in developing heart tissue and blood vessels, suggesting a role in cardiovascular system development [64,65]. Identifying the role of this gene in equine placenta angiogenesis could add valuable information to the reported genes already identified to have an effect in this process. In recent studies [68,69,70], several angiogenic genes (*ANGPT, VEGF, RTL1, VEGF, VEGFR1, ANGPT1,* and *eNOS)* have been identified to show different gene expression patterns which can have an effect on placental development, placental function, and fetal size.

When comparing the 4M to the 6M samples, we observed that the Pleckstrin Homology Like Domain Family A Member 2 (*PHLDA2*) had increased methylation and lower expression. *PHLDA2* has been identified to be maternally expressed in mouse placentae, and prior to the formation of the mature placenta, it is expressed in the yolk sac [71,72,73,74]. It is suggested that this gene modulates the accumulation of placental glycogen and placental growth, and the loss of function of this gene in mouse models was linked to an enlarged placenta and junctional zone and increased placental glycogen with no fetal overgrowth [71]. On the other hand, increased expression of this gene resulted in placental development inhibition, decreased placental glycogen, and loss of spongiotrophoblast lineage in mice, leading to asymmetric fetal growth restriction [71,75,76]. Studies researching the function of this gene have also identified its negative association with the expression of several placental hormones (i.e., placental prolactin and pregnancy-specific glycoproteins) [71]. In the human placenta, higher expression of this gene has been related to fetal growth restriction or low birthweight, reduced fetal movements, and placental weight [77]. These results shed light on the association of increased expression of this gene with poor perinatal outcomes [77]. This finding is remarkable due to the expression pattern we observed in our results, where this gene presented increased methylation and low expression, which would be necessary for normal placental and hormonal development in equine pregnancy.

Overall, our data demonstrated the importance of DNA methylation in placental development at the molecular level by profiling the placental methylome in the equine placenta for the first time. We identified a tendency that indicates that in genomic features with potential regulatory impact (promoters and introns) methylation increases towards the end of gestation, as has been reported in humans. Furthermore, we highlighted multiple biological pathways and genes related to placental development and function controlled by placental methylation. The provided information about the placental methylation patterns and their impact on gene expression serves as a foundation to further understand equine placental development during healthy pregnancy. The creation of our dataset is the crucial first step in identifying the role of altered methylation in placental pathologies.

## 4. Materials and Methods

### 4.1. Animal Use and Sample Collection

Previously sequenced samples [9,20] along with a new dataset were used in this study. The horses used for this study were mixed breed, weighed between 350 and 550 kg, and were four to nine years of age. The mares were kept in pasture and had access to water, minerals, and hay ad libitum. All animal procedures were approved by and were in accordance with the Institutional Animal Care and Use Committee of the University of Kentucky. The mares were bred naturally, and the gestational age was determined based on the day of ovulation (day 0). CA samples were collected from pregnant mares at four months (4M, n = 3), six months (6M, n = 3), and ten months of gestation (10M, n = 3), each sample from an individual dam. Briefly, the uterus of pregnant mares was recovered immediately after euthanasia (using a barbiturate overdose following the American Veterinary Medical Association (AVMA) guidelines for the euthanasia of animals), the CA was carefully separated from the endometrium, and full-thickness CA was collected from the body of the placenta (1 cm × 1 cm size), approximately 10 cm cranial to the cervical star. The collected samples were stored for 24 h at 4 °C in RNA*later*™ (Life Technologies, Carlsbad, CA, USA) and then stored at −80 °C. A second sample from the CA was fixed in formalin for 24 h and embedded in paraffin, and histological sections were stained with hematoxylin and eosin following standard procedures and examined to verify normal CA without any sign of inflammation and contamination from the endometrium.

### 4.2. DNA Methylome Analysis

#### 4.2.1. DNA Extraction, Library Preparation, and Sequencing

All the samples were thawed on ice, and the DNA was extracted using the Tissue DNA Extraction Kit (Qiagen, Gaithersburg, MD, USA), according to the manufacturer’s instructions. Following the extraction, the DNA obtained was analyzed using Qubit^®^ (Thermo Fisher Scientific, Waltham, MA, USA) and Bioanalyzer^®^ (Agilent, Santa Clara, CA, USA) to evaluate and determine concentration, purity, and integrity. For library preparation and sequencing, negative control DNA (lambda DNA) was added to the extracted DNA. Subsequently, the digestion of samples by methylation-insensitive restriction enzyme MspI was performed. At this point, size selection of DNA fragments with insertion lengths ranging from 40 bp to 220 bp was performed by gel cutting and the size-selected DNA fragments were bisulfite-treated (reduced representation bisulfite sequencing (RRBS)) using an EZ DNA Methylation Gold Kit (Zymo Research, Zymo Research, Orange, CA, USA). After this treatment, cytosines without methylation changed to uracil (after PCR amplification to thymidine), while cytosines with methylation remained unchanged [78]. The final DNA libraries were obtained by PCR amplification. Quality control was assessed for each library, and sequencing was performed on an Illumina HiSeq platform (Novogene, Sacramento, CA, USA).

#### 4.2.2. Data Processing and Bioinformatic Analysis

The raw data were evaluated using *fastqc* [79] to generate quality-control reports. The raw reads were trimmed with *TrimGalore* and mapped to the current equine genome (EquCab 3.0, ENSEMBL version (GCA_002863925.1; Jan 2018)) using *Bismark* [78]. After alignment to the reference genome, the *Bismark* algorithm was used to identify “true methylated sites”. To achieve this, methylated and unmethylated cytosines were counted at each site. Since the sequencing depth of each site is different, to identify true methylated sites, a one-tailed binomial test was performed at each site using the methylated (converted) and unmethylated counts to define the proportion of methylation at the site relative to that expected by random conversion of bases (H_0_: methylated C = unmethylated C = 0.5) [80,81]. Thresholds to properly identify methylated sites included: (1) a sequencing depth equal to or greater than five and (2) an FDR less than or equal to 0.01 [80,81]. Next, true methylated sites were annotated to the reference transcriptome (EquCab3.0 GTF version 104.3), and DNA methylation was considered to be associated with a specific gene when found 1 kb upstream or downstream of the gene TSS or the TTS, respectively [82,83].

The differential methylation analysis between different stages of pregnancy (4M, 6M, and 10M) included the estimation of overall methylation density between time points, methylation density per chromosome, methylation distribution on the functional gene regions (CpG islands, CpG shores, untranslated regions at 3′ and 5′, promoters, exons, introns, and repeats), methylation distribution up/downstream 1kb, and gene body. The normality of all variables was assessed using the Shapiro–Wilk test, and parametric or nonparametric comparisons were made accordingly. A Student’s *t*-test was used to compare the number of mCs identified between samples from different stages (4M, 6M, and 10M). Linear regression models were used to evaluate the relationship between the number of methylated cytosines and chromosome size. Dunn tests were used to compare the methylation levels across the different developmental stages (4M, 6M, and 10M) and analyses of variance (ANOVA) were performed to test between gene features (CpG islands, CpG shores, promoters, introns, exons, untranslated regions, and repeated elements). Lastly, gene enrichment analyses of genes carrying DMRs were conducted using KEGG [84,85,86] and PANTHER [87].

### 4.3. Transcriptome Analysis

#### 4.3.1. RNA Extraction, Library Preparation, and Sequencing

Previously generated RNA-seq data from the same samples (4M, 6M, and 10M) were used in this study (GSE108279 [20]). Briefly, CA samples were thawed on ice and total RNA was extracted with the RNeasy Mini Kit (Qiagen, Gaithersburg, MD, USA), following the manufacturer’s recommendations. Total RNA concentration was measured using a NanoDrop DP-1000 spectrophotometer (ThermoFisher Scientific, Waltham, MA, USA) and a Bioanalyzer (Agilent, Santa Clara, CA, USA) to evaluate concentration, purity, and integrity. All samples had a 230/260 ratio > 1.8, a 260/280 ratio > 2.0, and an RNA integrity number > 8.0. Library preparation was performed using the TruSeq Stranded mRNA Sample Prep Kit (Illumina, San Diego, CA, USA), as per the manufacturer’s instructions. Sequencing was performed on a HiSeq 4000 (Illumina) using a HiSeq 4000 sequencing kit version 1, generating 150 bp paired-end reads. Fastq files were generated and demultiplexed using *bcl2fastq* v2.17.1.14 Conversion Software (Illumina).

#### 4.3.2. Data Processing and Bioinformatic Analysis

Raw reads were processed using the elvers (https://github.com/dib-lab/elvers; version 0.1, 10.5281/zenodo.3345045; (accessed on 9 July 2022) pipeline by means of the above-mentioned reference genome and GFT annotations (EqCab3.0 version 104.3). The pipeline utilizes *fastqc*, *trimmomatic* [88], and *salmon* [89] to obtain the transcripts per kilobase million (TPM) for each annotated gene. Then, *DESeq2* [90] was used to extract differentially expressed genes (DEGs) between the different stages of pregnancy (4M, 6M, and 10M). Enrichment analyses of the DEGs in biological pathways were performed using PANTHER [43].

## Figures and Tables

**Table 1:** Overlap between differentially expressed genes (DEGs) and genes with differentially methylated regions (DMRs) across gestation.

Gene ID	Gene Name	Chr	Log2FC- Expression	Log2FC- Methylation	Context	Region	Comparison	Agreement between DEG and DMR
ENSECAG00000023637	*ADAM33*	22	−1.07	0.89	CG	Exon	4M vs. 6M	Yes
ENSECAG00000009251	*ATXN1*	20	1.11	−0.83	CG	Exon	4M vs. 6M	Yes
ENSECAG00000021591	*B4GALNT1*	6	−1.51	−0.87	CG	Exon	4M vs. 10M	No
ENSECAG00000008566	*CTSE*	5	−2.63	−2.79	CHH	Exon	4M vs. 6M	No
ENSECAG00000015010	*CYP4F124*	21	−2.69	−0.65	CG	Exon	4M vs. 10M	No
ENSECAG00000015010	*CYP4F124*	21	−2.69	−0.55	CG	Exon	4M vs. 6M	No
ENSECAG00000013832	*DES*	6	−1.70	1.27	CG	Exon	6M vs. 10M	Yes
ENSECAG00000022980	*ENTPD8*	25	−3.15	−1.29	CG	Exon	4M vs. 6M	No
ENSECAG00000020345	*ESPN*	2	1.11	1.32	CG	Exon	4M vs. 10M	No *
ENSECAG00000014851	*ILDR2*	5	1.02	−0.64	CG	Exon	4M vs. 10M	Yes
ENSECAG00000011659	*KIAA1549*	4	1.12	1.64	CG	Exon	4M vs. 6M	No *
ENSECAG00000014702	*MEDAG*	17	−1.47	−2.08	CG	Exon	4M vs. 10M	No
ENSECAG00000014702	*MEDAG*	17	−1.47	−1.84	CG	Exon	4M vs. 6M	No
ENSECAG00000024536	*NFE2*	6	1.19	0.90	CG	Exon	4M vs. 10M	No *
ENSECAG00000014030	*Novel gene*	3	−1.79	−2.74	CG	Exon	4M vs. 10M	No
ENSECAG00000029317	AKR7A3	PJAA01003681.1	−1.97	1.68	CG	Exon	4M vs. 6M	Yes
ENSECAG00000016720	*OBSCN*	14	−3.60	−0.35	CG	Exon	4M vs. 10M	No
ENSECAG00000016720	*OBSCN*	14	−3.60	0.48	CG	Exon	4M vs. 6M	Yes
ENSECAG00000013202	*PLIN1*	1	4.01	−2.73	CG	Exon	4M vs. 6M	Yes
ENSECAG00000017152	*RNF17*	17	−2.09	0.94	CG	Exon	4M vs. 10M	Yes
ENSECAG00000024853	*RSPO2*	9	−1.24	6.34	CHH	Exon	6M vs. 10M	Yes
ENSECAG00000019227	*SLC9A7*	X	1.71	0.87	CG	Exon	4M vs. 10M	No *
ENSECAG00000019227	*SLC9A7*	X	1.71	0.90	CG	Exon	4M vs. 6M	No *
ENSECAG00000001372	*SYN1*	X	1.75	−1.13	CG	Exon	4M vs. 10M	Yes
ENSECAG00000001372	*SYN1*	X	1.75	−1.37	CG	Exon	4M vs. 6M	Yes
ENSECAG00000020605	*TRIM2*	2	1.10	1.16	CG	Exon	6M vs. 10M	No *
ENSECAG00000007169	*TTC22*	2	−1.50	0.45	CG	Exon	6M vs. 10M	Yes
ENSECAG00000039959	*ZDBF2*	18	2.35	−6.15	CHH	Exon	4M vs. 10M	Yes
ENSECAG00000007090	*ZFR2*	7	−1.73	−0.68	CG	Exon	6M vs. 10M	No
ENSECAG00000000207	*ACTA1*	1	−2.89	−1.33	CG	Intron	4M vs. 6M	No
ENSECAG00000023637	*ADAM33*	22	−1.07	0.89	CG	Intron	4M vs. 6M	Yes
ENSECAG00000008835	*ANKRD44*	18	1.49	−2.27	CG	Intron	4M vs. 6M	Yes
ENSECAG00000008835	*ANKRD44*	18	1.61	2.10	CG	Intron	6M vs. 10M	No *
ENSECAG00000020314	*ANO1*	12	1.90	0.56	CG	Intron	4M vs. 10M	No *
ENSECAG00000020461	*BMPR1A*	1	1.49	−2.77	CG	Intron	4M vs. 6M	Yes
ENSECAG00000009553	*CADM3*	5	2.07	1.01	CG	Intron	6M vs. 10M	No *
ENSECAG00000010078	*CIT*	8	1.33	7.18	CHH	Intron	4M vs. 10M	No *
ENSECAG00000010078	*CIT*	8	1.33	5.01	CHH	Intron	4M vs. 6M	No *
ENSECAG00000016852	*CPT1A*	12	−1.88	0.94	CG	Intron	4M vs. 10M	Yes
ENSECAG00000008566	*CTSE*	5	−2.63	−2.79	CHH	Intron	4M vs. 6M	No
ENSECAG00000015010	*CYP4F124*	21	−2.69	−0.65	CG	Intron	4M vs. 10M	No
ENSECAG00000015010	*CYP4F124*	21	−2.69	−0.55	CG	Intron	4M vs. 6M	No
ENSECAG00000020795	*DAGLA*	12	1.28	1.02	CG	Intron	4M vs. 10M	No *
ENSECAG00000006857	*DCAF10*	25	1.02	0.99	CG	Intron	4M vs. 6M	No *
ENSECAG00000013582	*DHRS3*	2	−1.08	0.68	CG	Intron	4M vs. 10M	Yes
ENSECAG00000019565	*DLX5*	4	−1.39	−1.58	CG	Intron	4M vs. 10M	No
ENSECAG00000023607	*DOCK5*	2	1.37	0.68	CG	Intron	4M vs. 6M	No *
ENSECAG00000020345	*ESPN*	2	1.11	1.32	CG	Intron	4M vs. 10M	No *
ENSECAG00000000014	*GAS6*	17	−1.03	−0.82	CG	Intron	4M vs. 6M	No
ENSECAG00000001312	*GPR146*	13	−1.18	0.74	CG	Intron	4M vs. 6M	Yes
ENSECAG00000014851	*ILDR2*	5	1.02	−0.64	CG	Intron	4M vs. 10M	Yes
ENSECAG00000014968	*JMY*	14	1.73	0.86	CG	Intron	4M vs. 10M	No *
ENSECAG00000011659	*KIAA1549*	4	1.12	1.64	CG	Intron	4M vs. 6M	No *
ENSECAG00000039058	*KRT6C*	6	−5.81	−6.16	CHH	Intron	4M vs. 6M	No
ENSECAG00000020216	*KRT7*	6	−1.09	−2.73	CHH	Intron	4M vs. 10M	No
ENSECAG00000000296	*LAMC3*	25	1.18	−4.45	CHH	Intron	4M vs. 6M	Yes
ENSECAG00000021630	*LGSN*	20	−5.34	1.29	CG	Intron	4M vs. 10M	Yes
ENSECAG00000021583	*LMOD1*	30	−1.45	1.03	CG	Intron	4M vs. 6M	Yes
ENSECAG00000023118	*MCC*	14	1.26	−0.76	CG	Intron	4M vs. 6M	Yes
ENSECAG00000024512	TNFRSF10B	2	1.26	−4.21	CHH	Intron	4M vs. 10M	Yes
ENSECAG00000022376	*Novel gene*	18	1.52	1.66	CG	Intron	4M vs. 6M	No *
ENSECAG00000022376	*Novel gene*	18	1.52	1.06	CG	Intron	4M vs. 6M	No *
ENSECAG00000033604	SLC7A4	8	2.72	1.01	CG	Intron	4M vs. 6M	No *
ENSECAG00000022376	*Novel gene*	18	1.09	−1.47	CG	Intron	6M vs. 10M	Yes
ENSECAG00000018904	*NOXA1*	25	−1.77	−1.29	CG	Intron	4M vs. 6M	No
ENSECAG00000016720	*OBSCN*	14	−3.60	−0.35	CG	Intron	4M vs. 10M	No
ENSECAG00000020485	*PCOLCE2*	16	−1.11	−1.80	CG	Intron	6M vs. 10M	No
ENSECAG00000001688	*PCSK6*	1	−1.36	4.83	CHH	Intron	4M vs. 10M	Yes
ENSECAG00000001688	*PCSK6*	1	−1.36	3.39	CHH	Intron	4M vs. 6M	Yes
ENSECAG00000023890	*PRRX2*	25	−1.11	3.56	CHH	Intron	4M vs. 10M	Yes
ENSECAG00000023890	*PRRX2*	25	−1.11	3.37	CHH	Intron	4M vs. 6M	Yes
ENSECAG00000037450	*PTPRB*	6	1.13	−0.61	CG	Intron	6M vs. 10M	Yes
ENSECAG00000026963	*PTPRR*	6	−4.59	0.57	CG	Intron	4M vs. 6M	Yes
ENSECAG00000009250	*RAMP1*	6	−1.04	1.12	CG	Intron	4M vs. 10M	Yes
ENSECAG00000017152	*RNF17*	17	−2.09	0.94	CG	Intron	4M vs. 10M	Yes
ENSECAG00000020875	*RUNX2*	20	1.55	0.84	CG	Intron	4M vs. 10M	No *
ENSECAG00000019793	*SETBP1*	8	1.70	−1.03	CG	Intron	4M vs. 6M	Yes
ENSECAG00000009334	*SLC15A1*	17	−2.27	1.62	CG	Intron	6M vs. 10M	Yes
ENSECAG00000006302	*SLC25A29*	24	−1.29	−1.22	CG	Intron	4M vs. 6M	No
ENSECAG00000014155	*SMOC2*	31	−1.62	1.48	CG	Intron	6M vs. 10M	Yes
ENSECAG00000022037	*SOX9*	11	−1.91	1.26	CG	Intron	4M vs. 6M	Yes
ENSECAG00000015256	*SPAG9*	11	1.41	1.42	CG	Intron	4M vs. 10M	No *
ENSECAG00000008819	*TASOR2*	29	1.05	−0.94	CG	Intron	4M vs. 10M	Yes
ENSECAG00000008819	*TASOR2*	29	1.05	−1.01	CG	Intron	4M vs. 6M	Yes
ENSECAG00000008038	*TENM4*	7	1.03	0.74	CG	Intron	6M vs. 10M	No *
ENSECAG00000007718	*TMOD1*	25	−2.01	−6.62	CHH	Intron	6M vs. 10M	No
ENSECAG00000026887	*TSPAN8*	6	−1.54	−0.96	CG	Intron	6M vs. 10M	No
ENSECAG00000013193	*UHRF1*	7	1.37	−2.17	CG	Intron	4M vs. 10M	Yes
ENSECAG00000013193	*UHRF1*	7	1.37	−2.19	CG	Intron	4M vs. 6M	Yes
ENSECAG00000022984	*ZFHX3*	3	1.50	1.41	CG	Intron	6M vs. 10M	No *
ENSECAG00000007090	*ZFR2*	7	−1.73	−0.68	CG	Intron	6M vs. 10M	No
ENSECAG00000024955	*ZFYVE28*	3	−5.29	−2.03	CG	Intron	4M vs. 10M	No
ENSECAG00000016852	*CPT1A*	12	−1.88	1.53	CG	Promoter	4M vs. 10M	Yes
ENSECAG00000013582	*DHRS3*	2	−1.08	0.68	CG	Promoter	4M vs. 10M	Yes
ENSECAG00000012011	*FBN2*	14	1.43	−1.13	CG	Promoter	4M vs. 10M	Yes
ENSECAG00000012011	*FBN2*	14	1.43	−1.16	CG	Promoter	4M vs. 6M	Yes
ENSECAG00000001312	*GPR146*	13	−1.18	0.87	CG	Promoter	4M vs. 6M	Yes
ENSECAG00000016708	*HSF4*	3	−1.04	1.27	CG	Promoter	4M vs. 6M	Yes
ENSECAG00000016708	*HSF4*	3	−1.04	−1.45	CG	Promoter	6M vs. 10M	No
ENSECAG00000023496	*IRX3*	3	−5.44	1.57	CG	Promoter	4M vs. 6M	Yes
ENSECAG00000021630	*LGSN*	20	−5.34	1.29	CG	Promoter	4M vs. 10M	Yes
ENSECAG00000013691	*MALT1*	8	1.55	−5.04	CHH	Promoter	4M vs. 10M	Yes
ENSECAG00000033406	*Novel gene*	30	2.30	0.78	CG	Promoter	4M vs. 10M	No
ENSECAG00000029317	AKR7A3	PJAA01003681.1	−1.97	1.68	CG	Promoter	4M vs. 6M	Yes
ENSECAG00000000991	*PNMA3*	X	1.68	1.41	CG	Promoter	4M vs. 6M	No
ENSECAG00000000484	*PPARA*	28	1.11	−1.59	CG	Promoter	4M vs. 10M	Yes
ENSECAG00000024853	*RSPO2*	9	−1.24	6.34	CHH	Promoter	6M vs. 10M	Yes
ENSECAG00000001372	*SYN1*	X	1.75	−1.13	CG	Promoter	4M vs. 10M	Yes
ENSECAG00000001372	*SYN1*	X	1.75	−1.37	CG	Promoter	4M vs. 6M	Yes
ENSECAG00000008819	*TASOR2*	29	1.05	−0.94	CG	Promoter	4M vs. 10M	Yes
ENSECAG00000008819	*TASOR2*	29	1.05	−1.01	CG	Promoter	4M vs. 6M	Yes
ENSECAG00000001072	*TYMP*	28	−2.34	−1.16	CG	Promoter	6M vs. 10M	No
ENSECAG00000001072	*TYMP*	28	−2.34	−1.27	CG	Promoter	6M vs. 10M	No
ENSECAG00000013202	*PLIN1*	1	4.01	−2.73	CG	TES	4M vs. 6M	Yes
ENSECAG00000001372	*SYN1*	X	1.75	−1.13	CG	TSS	4M vs. 10M	Yes
ENSECAG00000001372	*SYN1*	X	1.75	−1.37	CG	TSS	4M vs. 6M	Yes
ENSECAG00000007090	*ZFR2*	7	−1.73	−0.68	CG	Utr3	6M vs. 10M	No
ENSECAG00000029317	AKR7A3	PJAA01003681.1	−1.97	1.68	CG	Utr5	4M vs. 6M	Yes
ENSECAG00000024853	*RSPO2*	9	−1.24	6.34	CHH	Utr5	6M vs. 10M	Yes

* positive correlation between the gene body methylation and gene expression.

## Data Availability

DNA sequencing and RRBS data have been deposited in the Sequence Read Archive (PRJNA541840 and PRJNA939133).

## Supplementary Materials

The following supporting information can be downloaded at: https://www.mdpi.com/article/10.3390/ijms24087084/s1.
