# Supplementary figures and images for: Dynamics of the Equine Placental DNA Methylome and Transcriptome from Mid- to Late Gestation
Source: Int J Mol Sci. 2023 Apr 11;24(8):7084. doi: 10.3390/ijms24087084 (PMC10139181; doi:10.3390/ijms24087084)

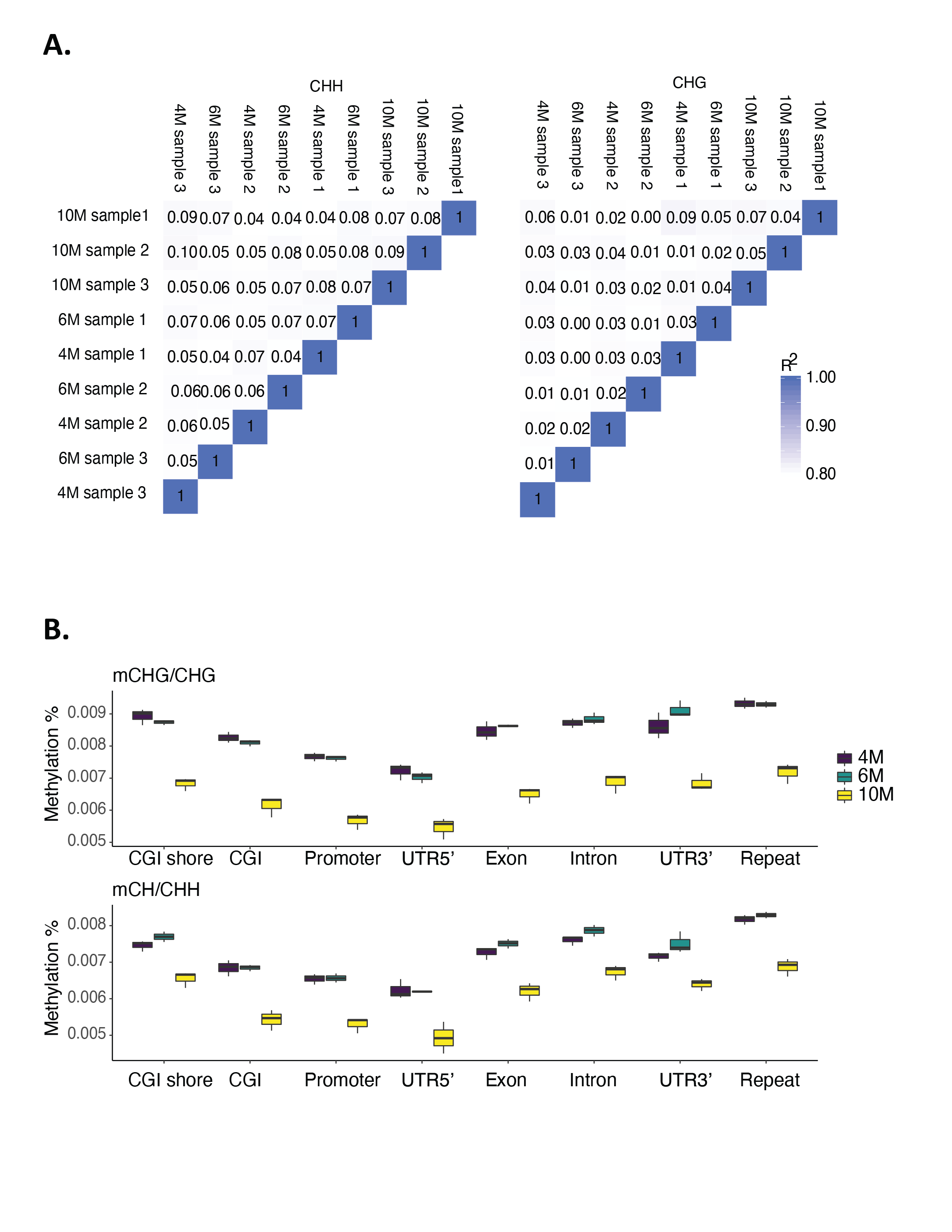

Supplement: Supplementary file 1 [file ijms-24-07084-s001.zip › Suplemmentary figures/SupFigure1.png]

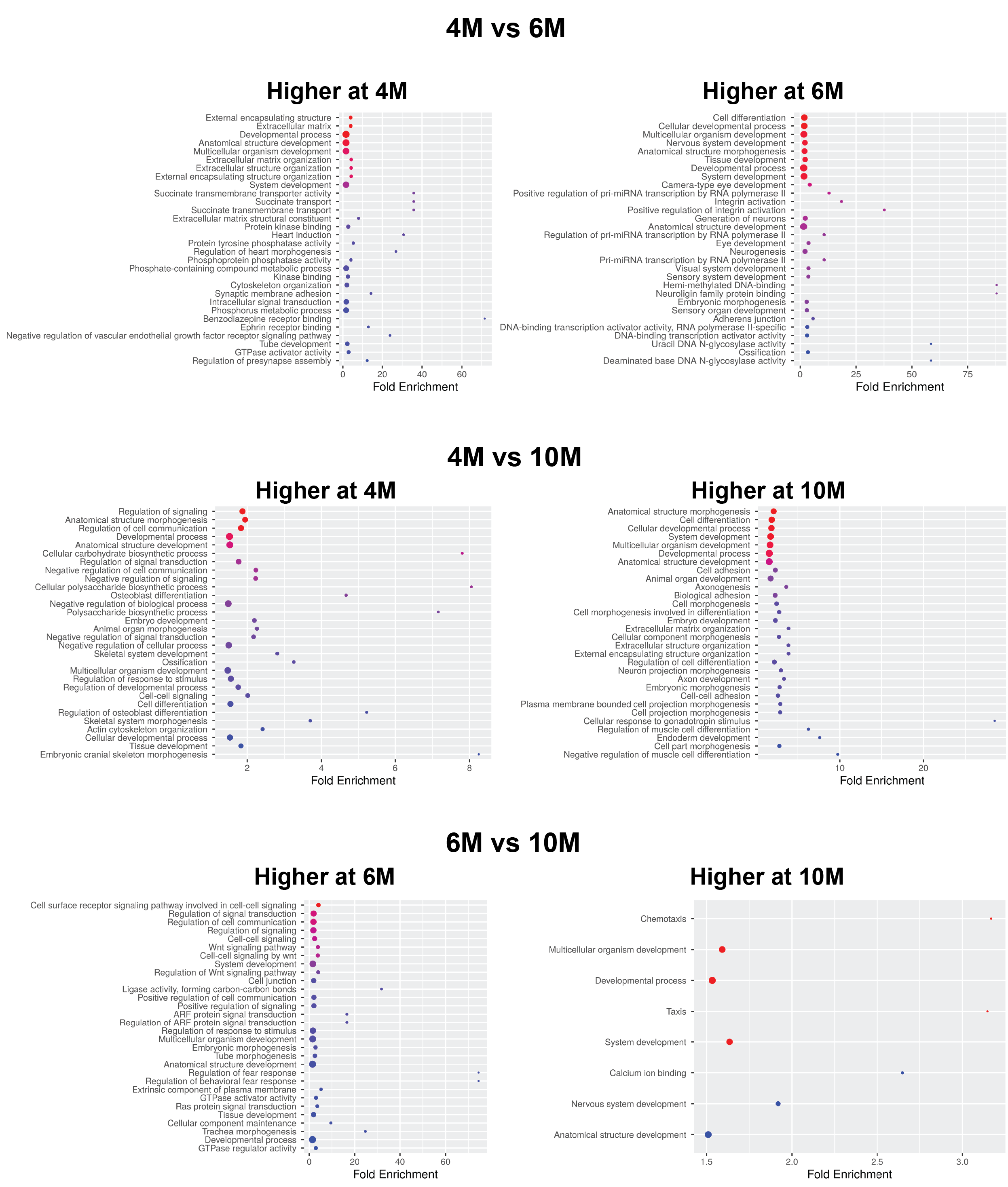

Supplement: Supplementary file 1 [file ijms-24-07084-s001.zip › Suplemmentary figures/SupFigure3.png]

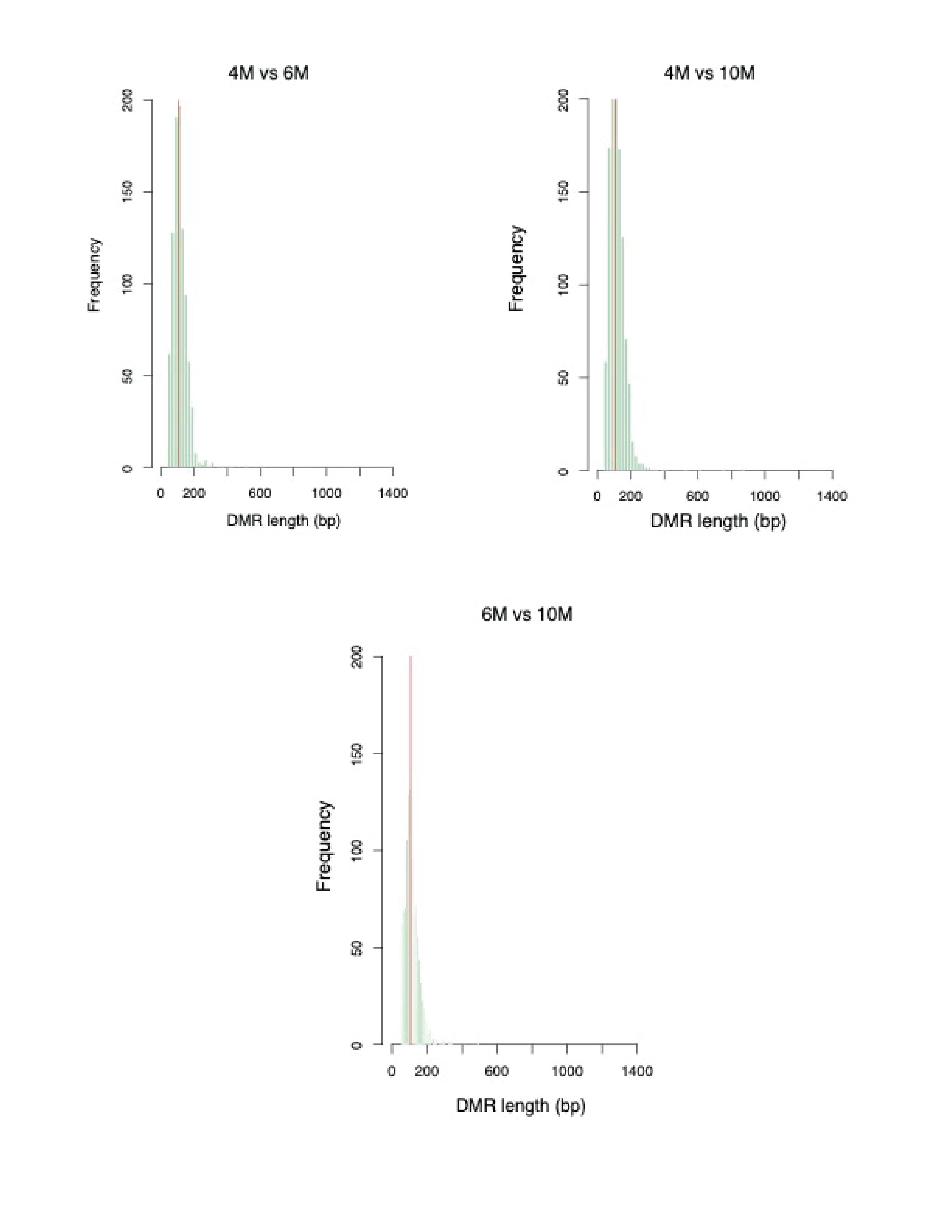

Supplement: Supplementary file 1 [file ijms-24-07084-s001.zip › Suplemmentary figures/SupFigure2.png]
